# Low anterior resection syndrome – what's the urgency?

**DOI:** 10.1111/ans.70116

**Published:** 2025-04-03

**Authors:** Kate Wilson, Michael Solomon, Julie A. Cornish, Aaron Quyn, Julie Croft, Andrew Stevenson, Andrea M. Warwick, Kheng‐Seong Ng

**Affiliations:** ^1^ Surgical Outcomes Research Centre (SOuRCe) Royal Prince Alfred Hospital (RPAH) Sydney New South Wales Australia; ^2^ Faculty of Medicine and Health, Central Clinical School The University of Sydney Sydney New South Wales Australia; ^3^ RPA Academic Institute of Surgery, Department of Colorectal Surgery Royal Prince Alfred Hospital Sydney New South Wales Australia; ^4^ University Hospital of Wales Healthcare NHS Trust Cardiff and Vale University Health Board Cardiff UK; ^5^ Leeds Institute of Medical Research at St James's University of Leeds Leeds UK; ^6^ Leeds Institute of Clinical Trials Research University of Leeds Leeds UK; ^7^ Colorectal Surgical Unit Royal Brisbane and Women's Hospital Brisbane Queensland Australia; ^8^ Department of Surgery University of Queensland Brisbane Queensland Australia; ^9^ Brisbane Academic Functional Colorectal Unit QEII Jubilee Hospital Brisbane Queensland Australia

With 15 400 cases p.a., colorectal cancer (CRC) is the fourth most commonly diagnosed cancer in Australia. The local age‐standardized mortality rate declined from 35 to 20 deaths per 100 000 people between 2000 and 2023. With improved survival outcomes, the focus has rightly shifted to survivorship after CRC surgery.^1^

Treatment of rectal and sigmoid cancer typically involves a combination of surgery, chemotherapy, radiotherapy and/or immunotherapy, all of which can affect cancer survivorship. Following a low anterior or abdominoperineal resection, sexual dysfunction has been reported in 71% of females and 97% of males^2^, ^3^ and urinary dysfunction reported in between 15% and25% of patients.^4^ Another well described sequala following anterior resection for sigmoid or rectal cancer is ‘low anterior resection syndrome’ (LARS).^5^, ^6^

LARS is constellation of bowel dysfunction symptoms following anterior resection, including faecal urgency, incontinence, bowel frequency, obstructive defecation, fragmentation and stool clustering. All can severely impact on quality of life.^7^ A validated, patient‐administered questionnaire measures defecatory dysfunction, generating a LARS Score to categorize patients as having ‘minor’ or ‘major LARS’.^8^

LARS prevalence ranges from 82% to 25% over assessment periods of 1–6 years.^9^, ^10^ A meta‐analysis using the LARS score questionnaire estimated the prevalence of major LARS at 41% based on 11 studies.^11^

With increasing survivorship, the need for greater understanding of the LARS self‐identification processes, diagnostic pathways, treatment efficacy, and availability brings with it a sense of urgency.

There are three main accepted treatment approaches for LARS.^12^ Supportive care includes dietary modifications, physiotherapy, and medication. If ineffective, more invasive treatments are considered. These include transanal irrigation (TAI) which empties the bowel by introducing water into the neorectum, and sacral neuromodulation (SNM) which delivers electrical impulses to the nerve(s) presumed to influence bowel control.

Evidence to support these treatments is poor. NICE Guidelines for Optimal Management of LARS^13^ noted the lack of randomized, comparative studies on treatment effectiveness. A systematic review further highlighted the need for well‐conducted, randomized multicentre trials.^14^

With poor evidence to support the various treatment options currently in use, what is next?

A UK NIHR & NHMRC collaborative grant scheme commissioned call for ‘Non‐conservative treatments for major low anterior resection syndrome’ has enabled Australian and UK colorectal surgeons to collaborate for the POLARiS Study, Pathway of Low Anterior Resection Syndrome Relief after Surgery.^15^, ^16^, ^17^

POLARiS is an international, multi‐arm, phase 3 randomized superiority trial (RCT) within a cohort (TWiCs) design aiming to determine the clinical and cost‐effectiveness of TAI or SNM versus optimized conservative management (OCM) which includes diet modification, physiotherapy, and medications. The cohort will describe the natural history of LARS and identify predictors of major LARS. Consumer engagement helped inform the design and confirmed the importance and relevance of study questions.

The authors of this Perspective are the POLARiS international leadership group which supports Australian and UK collaboration to deliver this definitive study. At least 20 UK hospitals will recruit 600 RCT and 1500 cohort participants and 16 Australian hospitals will recruit 200 and 500 participants respectively. Recruitment commenced in the UK in early 2024 and in Australia in late 2024.

The cohort enrolment is for adults who underwent an anterior resection within the past 10 years and have no bowel symptoms or minor LARS. The two‐year follow‐up will explore the natural history of LARS, identify predictors of major LARS, and screen participants for recruitment to the RCT. No treatment intervention is provided.

The RCT will evaluate the clinical and cost‐effectiveness and safety of TAI or SNM versus OCM for major LARS. Participants will be identified either in the clinic or from the cohort when symptoms escalate.

This collaboration has provided further opportunities for Australian researchers to complement trial activities with locally led research aiming to identify the challenges, barriers, and unmet needs in the management of LARS in Australia and New Zealand.

A survey of ANZ colorectal surgeons and Australian nurses and allied health staff will assess pre‐op education, screening and early management, views on treatment effectiveness and acceptability, healthcare resource utilization, barriers to treatment and perception of unmet needs of patients with LARS.

An assessment of 10 Australian Pelvic Floor or other specialist clinics will focus on evaluating current practices, treatment availability, and referral algorithms, and includes qualitative interviews of staff. Access to public and private health services, which can support timely diagnosis, can be delayed by a number of factors including prolonged wait times for clinical review, variability in local assessment and treatment options, and long waiting lists for conservative and non‐conservative treatment interventions.

A qualitative study with 24 POLARiS participants will explore lived experience, post‐surgery and cancer survivorship expectations, study treatment and participation acceptability. Interviews will also explore prior symptom management, treatment barriers, unmet needs, urinary and sexual function, and financial costs incurred by LARS, including impact on paid and unpaid work and educational opportunities.

With the impact on quality of life, social and work functioning, CRC survivors with LARS urgently want evidence‐based effective treatments, timely access to specialist clinics and access to affordable treatments, all of which have the potential to improve their cancer survivorship experience.

This review will provide insight into current treatment practices, preferences, and availability prior to the implementation of POLARiS results. We aim for our documentation of current practices, local challenges, barriers, and unmet needs, when combined with POLARiS study results, to support much needed critical change in the LARS treatment landscape in Australia and globally.

## Author contributions

**Kate Wilson**: Conceptualization; writing – original draft. **Michael Solomon**: Conceptualization; supervision; writing – review and editing. **Julie A. Cornish:** Conceptualization; writing – review and editing. **Aaron Quyn**: Conceptualization; writing – review and editing. **Julie Croft**: Conceptualization; writing – review and editing. **Andrew Stevenson**: Conceptualization; writing – review and editing. **Andrea M. Warwick:** Conceptualization; writing – review and editing. **Kheng‐Seong Ng**: Conceptualization; supervision; writing – review and editing.

## Conflicts of interest

None declared.
